# Overall and Cervical Cancer Survival in Patients With and Without Mental Disorders

**DOI:** 10.1001/jamanetworkopen.2023.36213

**Published:** 2023-09-29

**Authors:** Eva Herweijer, Jiangrong Wang, Kejia Hu, Unnur A. Valdimarsdóttir, Hans-Olov Adami, Pär Sparén, Karin Sundström, Fang Fang

**Affiliations:** 1 Institute of Environmental Medicine, Karolinska Institutet, Stockholm, Sweden; 2 Department of Laboratory Medicine, Karolinska Institutet, Stockholm, Sweden; 3 Centre of Public Health Sciences, Faculty of Medicine, University of Iceland, Reykjavik; 4 Department of Epidemiology, Harvard T.H. Chan School of Public Health, Boston, Massachusetts; 5 Department of Medical Epidemiology and Biostatistics, Karolinska Institutet, Stockholm, Sweden; 6 Clinical Effectiveness Group, Institute of Health and Society, University of Oslo, Oslo, Norway

## Abstract

**Importance:**

Individuals with a mental disorder experience substantial health disparity and are less likely to participate in cervical screening and human papillomavirus vaccination. Additionally, this population may benefit less from tertiary cancer prevention.

**Objective:**

To compare clinical characteristics and survival patterns between patients with cervical cancer with and without a preexisting diagnosis of a mental disorder at the time of cervical cancer diagnosis.

**Design, Setting, and Participants:**

This cohort study obtained data from Swedish population-based (Swedish Cancer Register, Swedish Cause of Death Register, Swedish Total Population Register, Swedish Patient Register, and Swedish Longitudinal Integration Database for Health Insurance and Labor Market Studies) and quality registries (Swedish Quality Register of Gynecologic Cancer and Swedish National Cervical Screening Register) on patients with cervical cancer. Patients who were included in the analysis were identified using the Swedish Cancer Register and were diagnosed with cervical cancer between 1978 and 2018. The Swedish Patient Register was used to identify patients with mental disorders using codes from the *International Classification of Diseases, Eighth Revision* and *Ninth Revision* and the *International Statistical Classification of Diseases and Related Health Problems, Tenth Revision.* Because data on clinical characteristics at the time of cancer diagnosis were available for only for part of the study population, 2 patient groups were created: those with cervical cancer diagnosed from 2002 to 2016 and all patients diagnosed with cervical cancer (1978-2018). Data analyses were carried out between March and September 2022.

**Exposure:**

Clinical diagnoses of a mental disorder, including substance abuse, psychotic disorders, depression, anxiety, stress-related disorders, attention-deficit/hyperactivity disorder, autism, and intellectual disability, prior to cervical cancer.

**Main Outcomes and Measures:**

Death due to any cause or due to cervical cancer as ascertained from the Swedish Cause of Death Register.

**Results:**

The sample included 20 177 females (mean [SD] age, 53.4 [17.7] years) diagnosed with cervical cancer from 1978 to 2018. In a subgroup of 6725 females (mean [SD] age, 52.2 [18.0] years) with cervical cancer diagnosed from 2002 to 2016, 893 (13.3%) had a preexisting diagnosis of a mental disorder. Compared with patients with no preexisting mental disorder diagnosis, those with a preexisting mental disorder had a higher risk of death due to any cause (hazard ratio [HR], 1.32; 95% CI, 1.17-1.48) and due to cervical cancer (HR, 1.23; 95% CI, 1.07-1.42). These risks were lower after adjustment for cancer characteristics at the time of cancer diagnosis (death due to any cause: HR, 1.19 [95% CI, 1.06-1.34] and death due to cervical cancer: HR, 1.12 [95% CI, 0.97-1.30]). Risk of death was higher for patients with substance abuse, psychotic disorders, or mental disorders requiring inpatient care. Among patients with cervical cancer diagnosed from 1978 to 2018, the estimated 5-year survival improved continuously during the study period regardless of preexisting diagnosis of a mental disorder status. For example, in 2018, the estimated 5-year overall survival proportion was 0.66 (95% CI, 0.60-0.71) and 0.74 (95% CI, 0.72-0.76) for patients with and without a preexisting diagnosis of a mental disorder, respectively.

**Conclusions and Relevance:**

Findings of this cohort study suggest that patients with cervical cancer and a preexisting diagnosis of a mental disorder have worse overall and cervical cancer–specific survival than patients without a preexisting mental disorder diagnosis, which may be partly attributable to cancer and sociodemographic characteristics at diagnosis. Hence, individuals with mental disorders deserve special attention in the tertiary prevention of cervical cancer.

## Key Points

**Question:**

What are the survival patterns of patients with cervical cancer with and without a preexisting diagnosis of a mental disorder?

**Findings:**

In this cohort study of 20 177 patients with cervical cancer diagnosed between 1978 and 2018, patients with a preexisting diagnosis of a mental disorder had a 32% higher risk of death due to any cause and a 23% higher risk of death due to cervical cancer. After adjustment for cancer characteristics at diagnosis, risks remained elevated at 19% and 12%, respectively; however, the difference in risk of death due to cervical cancer was no longer statistically significant.

**Meaning:**

Findings of this study suggest that patients with cervical cancer and a preexisting mental disorder diagnosis had higher overall and cervical cancer–specific mortality, a difference that may be partly attributable to cancer and sociodemographic characteristics at diagnosis.

## Teaser

This cohort study examines overall and cervical cancer–specific survival in patients with cervical cancer according to the presence or absence of a mental disorder diagnosis at the time of cancer diagnosis.

## Introduction

Cervical cancer is the fourth most common cancer among individuals globally.^1,2^ As the causal role of human papillomavirus (HPV) is well established,^3,4^ various measures have been implemented to prevent cervical cancer. Papanicolaou test screening to detect and treat precursor lesions has substantially reduced cervical cancer incidence,^5^ whereas HPV vaccination is expected to ultimately prevent the vast majority of cases.^6^ The World Health Organization issued a global strategy to eliminate cervical cancer as a public health problem by 2030.^7^ To succeed, high vaccination coverage and cervical screening uptake need to be achieved as well as early diagnosis and optimal management of invasive disease. Factors associated with cervical cancer outcomes include cancer stage at diagnosis, age at diagnosis, and tumor histologic type^8,9,10,11^; thus, early diagnosis and effective cancer treatment are the tertiary prevention methods available for reducing mortality due to cervical cancer among individuals who have invasive disease.^12^ Health inequities are, however, believed to pose one of the greatest challenges to meet the elimination goals.^13,14^

Mental illness constitutes a major cause of morbidity and mortality in today’s society.^15,16,17^ The burden is especially high among females, with the prevalence of any mental disorder estimated to be 12 760 per 100 000 females, and prevalence of anxiety and depressive disorders estimated to be 3779 and 3440 per 100 000 females, respectively.^18^ During the past decade, individuals with mental disorders have been recognized as experiencing substantial health disparity. With regard to cervical cancer prevention, females with mental illness are less likely to be vaccinated against HPV and to participate in cervical screening.^19,20,21,22^ Individuals with mental health issues may also benefit less from tertiary cervical cancer prevention due to delayed diagnosis of cancer and delayed start of treatment.^23,24^ For example, 1 study found that individuals with severe mental illness were diagnosed with cervical cancer at later stages and had a higher mortality rate after a cervical cancer diagnosis compared with those without severe mental illness.^25^

Because the existing literature on mental illness and cervical cancer outcomes is scarce, we compared clinical characteristics and survival of patients with cervical cancer with and without a preexisting diagnosis of a mental disorder using Swedish nationwide registers. As the study includes patients with cervical cancer diagnosed over 40 years, we also analyzed temporal changes in cervical cancer survival among patients with and without a diagnosed mental disorder at the time of cervical cancer diagnosis.

## Methods

### Study Population

This cohort study was approved by the Swedish Ethical Review Authority, which waived the need for written informed consent, as it is registry-based research. We followed the Strengthening the Reporting of Observational Studies in Epidemiology (STROBE) reporting guideline.

The study population comprised all individuals with a cervical cancer diagnosed between 1978 and 2018 as identified from the Swedish Cancer Register, which includes information on all malignant neoplasms diagnosed in Sweden since 1958.^26^ Using the national registration number assigned to all Swedish residents, we followed these individuals from the date of cancer diagnosis to the earliest date of death, emigration, or the end of the study period (December 31, 2019). Information on death and migration was taken from the Swedish Cause of Death Register and Swedish Total Population Register, respectively.

Detailed information on clinical characteristics at the time of cervical cancer diagnosis was available only for part of the study population. As such, 2 patient groups were created to fulfill different analytical purposes. The first group included all patients with cervical cancer diagnosed from 2002 to 2016 and was used to compare clinical characteristics at the time of cervical cancer diagnosis between patients with and without a preexisting diagnosis of a mental disorder. The 2002 to 2016 study period was chosen based on the availability of detailed clinical information in a nationwide case-control audit of cervical cancer, including a thorough clinical and histopathological review of all cervical cancer cases diagnosed from 2002 to 2011^27^ and the availability of clinical information in the nationwide Swedish Quality Register of Gynecologic Cancer from 2012 to 2016.^28^ The second group comprised all females diagnosed with cervical cancer from 1978 to 2018 and was used to compare trends in overall mortality as well as cervical cancer–related mortality over 40 years between individuals with and without a preexisting diagnosis of a mental disorder (eFigure in Supplement 1).

### Mental Disorder Diagnosis

Clinical diagnosis of a mental disorder prior to the diagnosis of cervical cancer was identified from the Swedish Patient Register, which includes largely complete data on inpatient care of mental disorders since 1973 and outpatient care of mental disorders since 2001, using codes from Swedish revisions of the *International Classification of Diseases, Eighth Revision* and *Ninth Revision *(*ICD-8* and *ICD-9,* respectively) and the *International Statistical Classification of Diseases and Related Health Problems, Tenth Revision *(*ICD-10*) (eTable 1 in Supplement 1). We included patients with psychiatric disorders, such as substance abuse (tobacco, alcohol, opioids, cannabinoids, sedatives or hypnotics, cocaine, other stimulants including caffeine, hallucinogens, volatile solvents, and other psychoactive substances), psychotic disorders, depression, anxiety, and stress-related disorders, as well as neurodevelopmental disorders, such as attention-deficit/hyperactivity disorder, autism spectrum disorder, and intellectual disability. We examined all mental disorders collectively as any mental disorder as well as by specific diagnosis. Primary and secondary diagnoses were considered when we ascertained mental disorders from the Swedish Patient Register. Due to the lack of registry data on outpatient care for mental disorders before 2001, clinical diagnoses of mental disorders were based on both inpatient and outpatient care in the 2002 to 2016 patient cohort but only on inpatient care in the 1978 to 2018 patient cohort.

### Outcomes

Study outcomes were death due to any cause (ie, overall mortality) and death from cervical cancer (ie, cervical cancer–specific mortality), as documented in the Swedish Cause of Death Register. Deaths documented as due to cervical cancer were identified if the underlying cause of death was listed as *ICD-8* or *ICD-9* code 180 or *ICD-10* code C53.

### Clinical Characteristics

For the group diagnosed with cervical cancer from 2002 to 2016, information on FIGO tumor stage (International Federation of Gynecology and Obstetrics staging system; stages IA, IB, or II or greater, unknown, or missing), tumor histologic type (squamous cell cancer, adenocarcinoma, or other [eg, adenosquamous cell carcinoma, glassy cell carcinoma, clear cell carcinoma, small cell carcinoma, large cell carcinoma, neuroendocrine carcinoma, and undifferentiated carcinoma]), and mode of detection (symptomatic or screen detected) was obtained from a case-control audit of cervical cancer^27^ and the Swedish Quality Register of Gynecologic Cancer.^28^ When data on mode of cancer detection were missing, the Swedish National Cervical Screening Register^29^ was used with an algorithm in which individuals with an abnormal Papanicolaou test result 1 to 12 months prior to cancer diagnosis were considered to have cancer detected on screening. For the group diagnosed with cervical cancer from 1978 to 2018, information on tumor histologic type was obtained from the Swedish Quality Register of Gynecologic Cancer by using the tumor pathological diagnosis code.

### Covariables

Information on region of residence at cancer diagnosis and year of birth was collected from the Swedish Total Population Register. Information on the highest educational attainment level (low, middle, high, or missing) and marital status (cohabiting, noncohabiting, or missing) at cancer diagnosis was obtained from the Swedish Longitudinal Integration Database for Health Insurance and Labor Market Studies. Educational levels were defined as low (less than high school), middle (high school), and high (university studies). Finally, the Swedish adaptation of the Charlson Comorbidity Index (CCI) score based on clinical diagnoses was calculated during the 12 months prior to cancer diagnosis using the inpatient and outpatient diagnoses retrieved from the Swedish Patient Register and scored as mild (CCI of 1 or 2) and moderate or severe (CCI ≥3).^30^

### Statistical Analysis

In the patient group diagnosed with cervical cancer from 2002 to 2016, we stratified clinical characteristics and other covariables by status of preexisting diagnosis of a mental disorder. The association of a preexisting diagnosis of a mental disorder with overall and cervical cancer–specific mortality was assessed using Cox proportional hazards models with multivariable adjustment, estimating hazard ratios (HRs) and 95% CIs. Crude incidence rates of overall and cervical cancer–specific death were calculated according to preexisting mental disorder diagnosis status and were reported per 1000 person-years. Time since cancer diagnosis was treated as the underlying time scale. Any preexisting diagnosis of a mental disorder was treated as the main exposure, whereas death due to any cause and death due to cervical cancer were treated as the main outcomes. A minimally adjusted model was fitted with further adjustment for calendar year of cancer diagnosis as a continuous covariable and age at cancer diagnosis as a categorical covariable (<30, 30-39, 40-49, 50-65, or >65 years). In a second model, further adjustment was made for additional covariables, including region of residence, educational level, marital status, and CCI, as well as mode of detection, FIGO score, and tumor histopathological subtype. We fitted these 2 models to estimate the HRs describing the extent to which the association of preexisting diagnosis of a mental disorder with overall as well as cervical cancer–specific mortality could be attributed to clinical and other characteristics at the time of cervical cancer diagnosis. Schoenfeld residuals of all variables were plotted by time to test for the assumption of proportional hazard. By visual check, we found the residuals to scatter independently of time (ie, to distribute randomly over time) and tests of nonzero slope of the residuals over time also argued against major violations of the assumption. Variables were subsequently adjusted for by stratification as needed. We studied the severity of mental disorders by classifying them as attended to solely via outpatient care or attended to via inpatient care (with or without outpatient care).

In the patient group diagnosed with cervical cancer between 1978 and 2018, we investigated temporal trends in overall and cervical cancer–specific survival by preexisting diagnosis of a mental disorder status using Cox proportional hazards models with multivariable adjustment, including calendar year of cancer diagnosis (10-year categories), age at diagnosis, region of residence, and tumor histopathological subtype. We used the log likelihood ratio test to assess the interaction between calendar year of cancer diagnosis and preexisting diagnosis of a mental disorder status. Although we used Cox proportional hazards models in the main analysis, assuming a constant magnitude of the associations during follow-up, we also used flexible parametric survival models to assess in more detail changes in the associations of preexisting diagnosis of a mental disorder with mortality, including a cubic spline for calendar year of cancer diagnosis and an interaction between the spline terms and preexisting diagnosis of a mental disorder status. We further modeled the age-standardized 5-year overall and cervical cancer–specific survival among patients with and without a preexisting diagnosis of a mental disorder. The age distribution of the person-years attributed by all patients in the cohort during follow-up was taken as the standard population.

Data management was done in SAS statistical software, version 9.4 (SAS Institute Inc), and statistical analyses were carried out in Stata, version 17 (StataCorp LLC). The threshold for statistical significance was a 2-sided *P* < .05. Data were analyzed between March and September 2022.

## Results

The sample included 20 177 females (mean [SD] 53.4 [17.7] years) diagnosed with cervical cancer from 1978 to 2018. In a subgroup of 6725 females (mean [SD] age, 52.2 [18.0] years) diagnosed with cervical cancer from 2002 to 2016, 893 (13.28%) had a preexisting diagnosis of a mental disorder. Patients with a preexisting diagnosis of a mental disorder were generally younger at cancer diagnosis, more often had squamous cell cancer, a lower-level of educational attainment, and lived alone compared with patients without a preexisting mental disorder diagnosis (Table 1). Patients requiring inpatient care for a mental disorder more often presented with FIGO stage II or greater disease at diagnosis compared with patients without a mental disorder diagnosis (eTable 2 in Supplement 1 ).

**Table 1.** Characteristics at Cervical Cancer Diagnosis in the 2002-2016 Patient Cohort Stratified by Presence of a Preexisting Diagnosis of a Mental Disorder

Characteristic	Patients, No. (%)			*P* value^a^
	Total	No mental disorder	Any mental disorder	
Participants	6725 (100)	5832 (86.7)	893 (13.28)	NA
Type of mental disorder				
Substance abuse^b^	313 (4.65)	NA	313 (35.05)	NA
Psychotic disorders^c^	92 (1.37)	NA	92 (10.30)	NA
Depressive disorder	392 (5.83)	NA	392 (43.90)	NA
Anxiety disorder	312 (4.64)	NA	312 (34.94)	NA
Stress-related disorders	258 (3.84)	NA	258 (28.89)	NA
Attention-deficit/hyperactivity disorder	50 (0.74)	NA	50 (5.60)	NA
Autism spectrum disorder	9 (0.13)	NA	9 (1.01)	NA
Intellectual disability	13 (0.19)	NA	13 (1.46)	NA
Year				
2002-2004	1268 (18.86)	1172 (20.10)	96 (10.75)	<.001
2005-2009	2148 (31.94)	1920 (32.92)	228 (25.53)	
2010-2014	2244 (33.37)	1878 (32.20)	366 (40.99)	
2015-2016	1065 (15.84)	862 (14.78)	203 (22.73)	
Age, mean (SD), y	52.21 (18.00)	52.51 (18.11)	50.29 (17.18)	<.001
<30	502 (7.46)	425 (7.29)	77 (8.62)	.02
30-39	1550 (23.05)	1321 (22.65)	229 (25.64)	
40-49	1415 (21.04)	1238 (21.23)	177 (19.82)	
50-65	1493 (22.20)	1283 (22.00)	210 (23.52)	
>65	1765 (26.25)	1565 (26.83)	200 (22.40)	
CCI, mean (SD)	0.77 (1.25)	0.77 (1.25)	0.82 (1.29)	.23
No comorbidities	4453 (66.22)	3878 (66.50)	575 (64.39)	.008
Mild (score 1-2)	1869 (27.79)	1625 (27.86)	244 (27.32)	
Moderate or severe (score ≥3)	403 (5.99)	329 (5.64)	74 (8.29)	
FIGO stage				
IA	1477 (21.96)	1272 (21.81)	205 (22.96)	.12
IB	2587 (38.47)	2257 (38.70)	330 (36.95)	
II or greater	2577 (38.32)	2237 (38.36)	340 (38.07)	
Unknown or missing	84 (1.25)	66 (1.13)	18 (2.02)	
Mode of cancer detection				
Screen-detected	2360 (35.09)	2039 (34.96)	321 (35.95)	.57
Symptomatic	4365 (64.91)	3793 (65.04)	572 (64.05)	
Tumor histologic type				
SCC	4831 (71.84)	4124 (70.71)	707 (79.17)	<.001
AC	1387 (20.62)	1260 (21.60)	127 (14.22)	
Other^d^	501 (7.45)	443 (7.60)	58 (6.49)	
Unknown or missing	6 (0.09)	5 (0.09)	1 (0.11)	
Region				
East	2765 (41.12)	2381 (40.83)	384 (43.00)	.42
South	2761 (41.06)	2395 (41.07)	366 (40.99)	
North	1190 (17.70)	1048 (17.97)	142 (15.90)	
Unknown or missing	9 (0.13)	8 (0.14)	1 (0.11)	
Educational level^e^				
Low	1667 (24.79)	1399 (23.99)	268 (30.01)	<.001
Medium	2970 (44.16)	2562 (43.93)	408 (45.69)	
High	1978 (29.41)	1772 (30.38)	206 (23.07)	
Unknown or missing	110 (1.64)	99 (1.70)	11 (1.23)	
Marital status				
Cohabiting	2441 (36.30)	2219 (38.05)	222 (24.86)	<.001
Noncohabiting	4283 (63.69)	3612 (61.93)	671 (75.14)	
Unknown or missing	1 (0.01)	1 (0.02)	0	

Abbreviations: AC, adenocarcinoma; CCI, Charlson Comorbidity Index; FIGO, International Federation of Gynecology and Obstetrics; NA, not applicable; SCC, squamous cell cancer.

^a^ *P* values for categorical variables were based on χ^2^ test, whereas *P* values for continuous variables were based on 2-sample *t* test.

^b^ Including tobacco, alcohol, opioids, cannabinoids, sedatives or hypnotics, cocaine, other stimulants including caffeine, hallucinogens, volatile solvents, and other psychoactive substances.

^c^ Including schizophrenia and nonaffective psychotic disorders.

^d^ Other tumor types include adenosquamous cell carcinoma, glassy cell carcinoma, clear cell carcinoma, small cell carcinoma, large cell carcinoma, neuroendocrine carcinoma, and undifferentiated carcinoma.

^e^ Educational levels defined as low (less than high school), middle (high school), or high (university studies).

In the subgroup of patients diagnosed with cervical cancer from 2002 to 2016, there were 2466 deaths due to any cause, of which 338 were among patients with a preexisting diagnosis of a mental disorder. The risk of death due to any cause was higher among patients with a mental disorder vs those without, in both partially adjusted (HR, 1.32; 95% CI, 1.17-1.48) and fully adjusted (HR, 1.19; 95% CI, 1.06-1.34) models (Table 2). Analyses stratified by type of mental disorders (Table 2) showed a higher risk of death in patients with substance abuse (partially adjusted model: HR, 1.52 [95% CI, 1.28-1.80]; fully adjusted model: HR, 1.27 [95% CI, 1.06-1.52]), psychotic disorders (partially adjusted model: HR, 1.74 [95% CI, 1.34-2.27]); fully adjusted model: HR, 1.48 [95% CI, 1.12-1.96]), and depressive disorders (partially adjusted model: HR, 1.26 [95% CI, 1.06-1.50]); fully adjusted model: HR, 1.25 [95% CI, 1.04-1.50]). Intellectual disability was not associated with risk of death after full adjustment. Anxiety and stress-related disorders were not associated with risk of death after partial and full data adjustment. A higher risk of death due to any cause was associated with mental disorders that required inpatient care (fully adjusted HR, 1.24; 95% CI, 1.09-1.42) but not with mental disorders that required only outpatient care (fully adjusted HR, 1.03; 95% CI, 0.81-1.32) (eTable 3 in Supplement 1).

**Table 2.** Overall Mortality in Patients With Cervical Cancer Diagnosed From 2002 to 2016 Stratified by Presence of a Preexisting Mental Disorder Diagnosis

	No. of deaths	No. of person-years	Crude IR per 1000 person-years (95% CI)	HR (95% CI)	
				Partially adjusted^a^	Fully adjusted^b^
Any mental disorder					
No	2128	42 148	50.49 (48.39-52.68)	1 [Reference]	1 [Reference]
Yes	338	5231	64.61 (58.08-71.88)	1.32 (1.17-1.48)	1.19 (1.06-1.34)
By type of psychiatric disorders					
Substance abuse^c^					
No	2329	45 684	50.98 (48.95-53.09)	1 [Reference]	1 [Reference]
Yes	137	1695	80.80 (68.34-95.53)	1.52 (1.28-1.80)	1.27 (1.06-1.52)
Psychotic disorders^d^					
No	2409	46 930	51.33 (49.32-53.42)	1 [Reference]	1 [Reference]
Yes	57	449	126.85 (97.85-164.45)	1.74 (1.34-2.27)	1.48 (1.12-1.96)
Depressive disorder					
No	2330	45 081	51.68 (49.63-53.83)	1 [Reference]	1 [Reference]
Yes	136	2299	59.16 (50.01-69.99)	1.26 (1.06-1.50)	1.25 (1.04-1.50)
Anxiety disorder					
No	2377	45 542	52.19 (50.14-54.33)	1 [Reference]	1 [Reference]
Yes	89	1838	48.43 (39.34-59.61)	1.15 (0.92-1.42)	1.13 (0.91-1.41)
Stress-related disorders					
No	2396	45 717	52.41 (50.35-54.55)	1 [Reference]	1 [Reference]
Yes	70	1663	42.10 (33.31-53.22)	1.03 (0.81-1.31)	0.95 (0.74-1.22)
By type of neurodevelopmental disorders					
Attention-deficit/hyperactivity disorder					
No	2458	47 120	52.16 (50.14-54.27)	1 [Reference]	1 [Reference]
Yes	8	259	30.84 (15.42-61.67)	1.33 (0.66-2.67)	1.07 (0.52-2.19)
Autism spectrum disorder					
No	2465	47 335	52.08 (50.06-54.17)	1 [Reference]	1 [Reference]
Yes	1	45	22.11 (3.11-156.97)	0.80 (0.11-5.70)	1.02 (0.14-7.67)
Intellectual disability					
No	2460	47 332	51.97 (49.96-54.07)	1 [Reference]	1 [Reference]
Yes	6	48	124.84 (56.09-277.89)	2.38 (1.07-5.30)	1.32 (0.57-3.07)

Abbreviations: HR, hazard ratio; IR, incidence rate.

^a^ Adjusted for age and calendar year of cervical cancer diagnosis (continuous variables).

^b^ Adjusted for age and calendar year of cervical cancer diagnosis (continuous variables) as well as FIGO (International Federation of Gynecology and Obstetrics) stage, tumor histologic type, residing region, educational level, and marital status.

^c^ Including tobacco, alcohol, opioids, cannabinoids, sedatives or hypnotics, cocaine, other stimulants including caffeine, hallucinogens, volatile solvents, and other psychoactive substances.

^d^ Including schizophrenia and nonaffective psychotic disorders.

Among patients diagnosed with cervical cancer from 2002 to 2016, there were 1625 deaths due to cervical cancer during follow-up, of which 224 occurred among patients with a preexisting diagnosis of a mental disorder. The risk of death due to cervical cancer was higher among patients with a preexisting mental disorder compared with those without such a disorder, after adjustment for age and year of diagnosis (HR, 1.23; 95% CI, 1.07-1.42), but this finding was not statistically significant in the fully adjusted model (HR, 1.12; 95% CI, 0.97-1.30). Analyses stratified by type of mental disorder showed a higher risk of death due to cervical cancer among patients with substance abuse (HR, 1.43; 95% CI, 1.16-1.77) and psychotic disorders (HR, 1.64; 95% CI, 1.18-2.30) after adjustment for age and year of cervical cancer diagnosis. The findings were no longer statistically significant when the models were further adjusted for clinical characteristics and other covariables (Table 3). Results stratified by type of care for mental disorder showed a higher risk of death due to cervical cancer associated with mental disorders requiring inpatient care (fully adjusted HR, 1.19; 95% CI, 1.01-1.40), but not with mental disorders requiring only outpatient care (fully adjusted HR, 0.94; 95% CI, 0.70-1.25) (eTable 4 in Supplement 1).

**Table 3.** Cervical Cancer–Specific Mortality Among Patients With Cervical Cancer Diagnosed From 2002 to 2016 Stratified by Presence of a Preexisting Mental Disorder Diagnosis

	No. of deaths	No. of person-years	Crude IR per 1000 person-years (95% CI)	HR (95% CI)	
				Partially adjusted^a^	Fully adjusted^b^
Any mental disorder					
No	1401	42 148	33.24 (31.54-35.03)	1 [Reference]	1 [Reference]
Yes	224	5231	42.82 (37.56-48.81)	1.23 (1.07-1.42)	1.12 (0.97-1.30)
By type of psychiatric disorders					
Substance abuse^c^					
No	1534	45 684	33.58 (31.94-35.3)	1 [Reference]	1 [Reference]
Yes	91	1695	53.67 (43.7-65.91)	1.43 (1.16-1.77)	1.21 (0.98-1.51)
Psychotic disorders^d^					
No	1590	46 930	33.88 (32.25-35.59)	1 [Reference]	1 [Reference]
Yes	35	449	77.89 (55.93-108.49)	1.64 (1.18-2.30)	1.37 (0.97-1.94)
Depressive disorder					
No	1538	45 081	34.12 (32.45-35.86)	1 [Reference]	1 [Reference]
Yes	87	2299	37.85 (30.67-46.70)	1.13 (0.91-1.40)	1.13 (0.91-1.42)
Anxiety disorder					
No	1568	45 542	34.43 (32.77-36.18)	1 [Reference]	1 [Reference]
Yes	57	1838	31.02 (23.92-40.21)	0.97 (0.75-1.27)	1.00 (0.76-1.32)
Stress-related disorders					
No	1577	45 717	34.49 (32.83-36.24)	1 [Reference]	1 [Reference]
Yes	48	1663	28.87 (21.76-38.31)	0.96 (0.72-1.28)	0.90 (0.67-1.20)
By type of neurodevelopmental disorders					
Attention-deficit/hyperactivity disorder					
No	1618	47 120	34.34 (32.7-36.05)	1 [Reference]	1 [Reference]
Yes	7	259	26.99 (12.87-56.61)	1.26 (0.60-2.66)	1.14 (0.53-2.45)
Autism spectrum disorder					
No	1625	47 335	34.33 (32.7-36.04)	1 [Reference]	1 [Reference]
Yes	0	45	NA	NA	NA
Intellectual disability					
No	1621	47 332	34.25 (32.62-35.96)	1 [Reference]	1 [Reference]
Yes	4	48	83.23 (31.24-221.76)	2.16 (0.81-5.77)	1.13 (0.40-3.15)

Abbreviations: HR, hazard ratio; IR, incidence rate; NA, not applicable.

^a^ Adjusted for age and calendar year of diagnosis (continuous variables).

^b^ Adjusted for age and calendar year of diagnosis (continuous) as well as FIGO (International Federation of Gynecology and Obstetrics) stage, tumor histologic type, region of residence, educational level, and marital status.

^c^ Includes tobacco, alcohol, opioids, cannabinoids, sedatives or hypnotics, cocaine, other stimulants including caffeine, hallucinogens, volatile solvents, and other psychoactive substances.

^d^ Including schizophrenia and nonaffective psychotic disorders.

**Figure 1.**
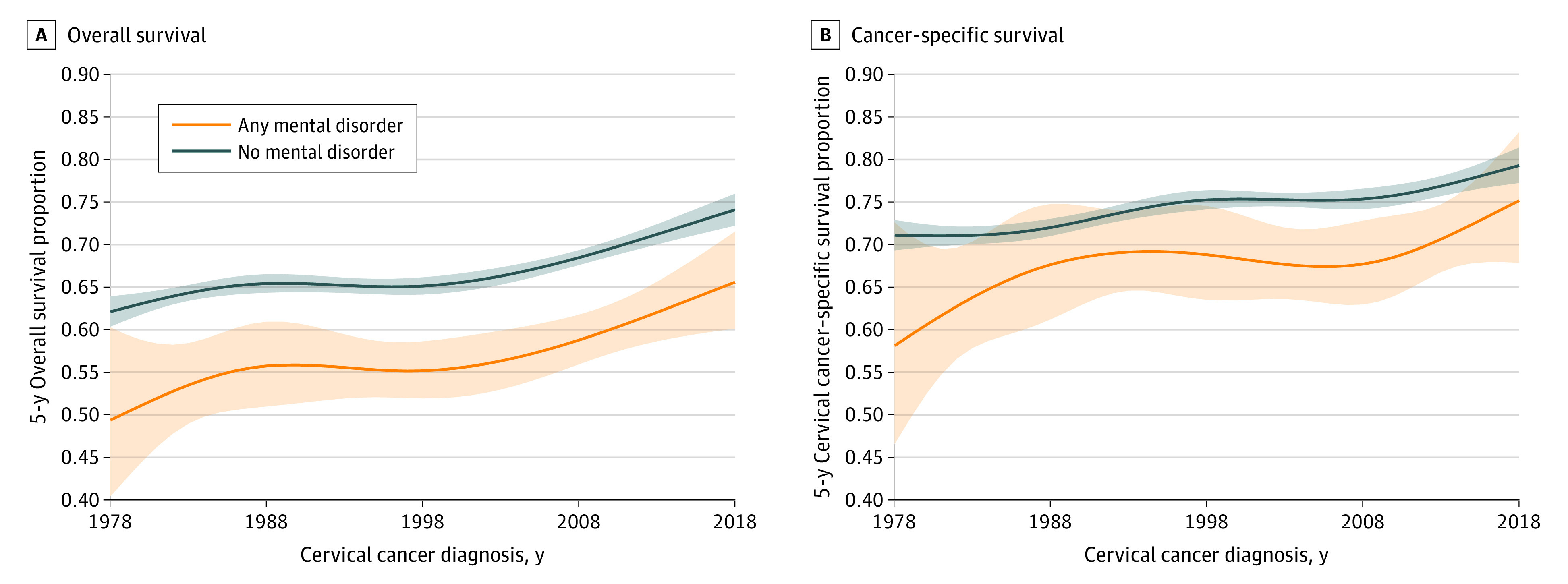
Comparison of Overall and Cervical Cancer–Specific Survival in Patients With and Without a Mental Disorder Five-year overall and cervical cancer–specific survival standardized to the age distribution of person-years attributed to all patients during follow-up and stratified by the presence of preexisting diagnosis of a mental disorder status and calendar year of diagnosis.

**Figure 2.**
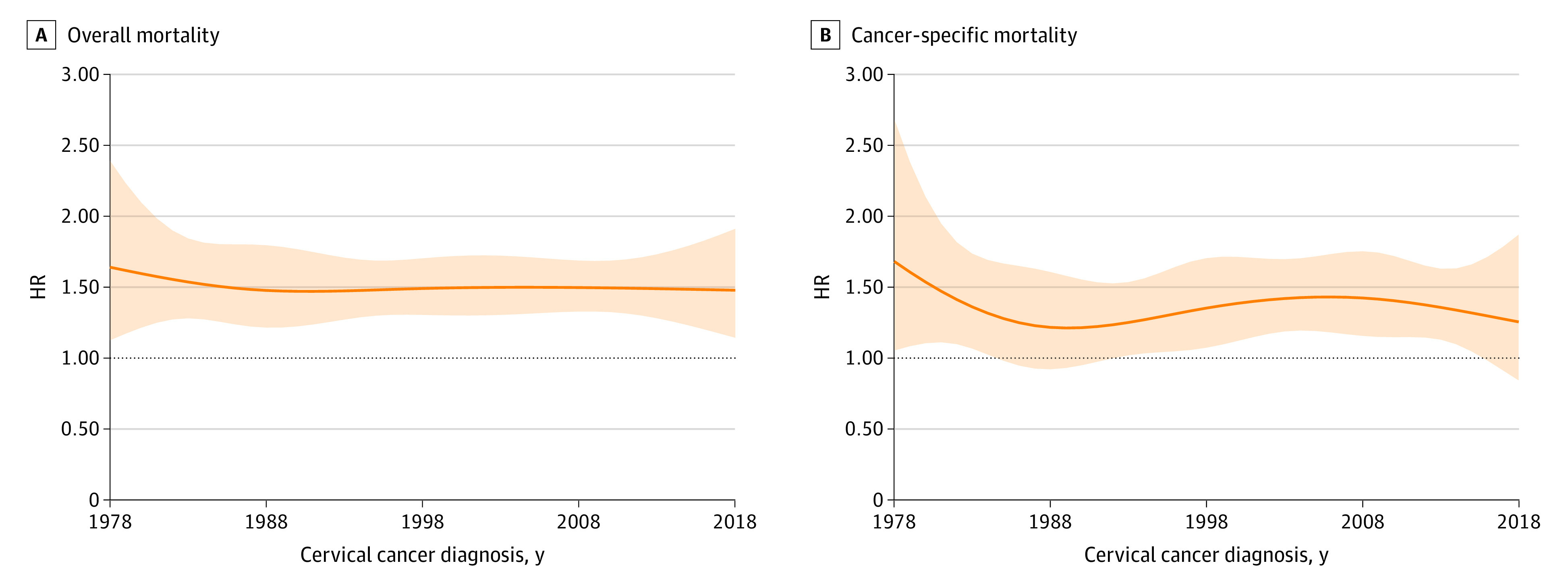
Risk of Overall and Cervical Cancer–Specific Mortality in Patients Diagnosed With Cervical Cancer From 1978 to 2018 Stratified by Calendar Year of Cancer Diagnosis Shaded area represents 95% CIs. HR indicates hazard ratio.

Improvements were observed in estimated 5-year overall and cervical cancer–specific survival during the study period, regardless of a preexisting diagnosis of a mental disorders (Figure 1). For example, between 1978 and 2018, the estimated 5-year overall survival proportion increased from 0.49 (95% CI, 0.40-0.60) to 0.66 (95% CI, 0.60-0.71) and from 0.62 (95% CI, 0.60-0.64) to 0.74 (95% CI, 0.72-0.76) for patients with and without a preexisting diagnosis of a mental disorder, respectively. We observed HRs of 1.53 (95% CI, 1.42-1.66) and 1.34 (95% CI, 1.20-1.49) when comparing risk of death due to any cause or due to cervical cancer between individuals with and without a preexisting diagnosis of a mental disorder, respectively (eTables 5 and 6 in Supplement 1). A consistently higher risk of overall mortality was found among patients with a preexisting mental disorder diagnosis than those without in analyses stratified by calendar year of cervical cancer diagnosis (Figure 2). For example, in 2018, HRs of 1.48 (95% CI, 1.14-1.91) and 1.25 (95% CI, 0.84-1.86) were observed when comparing the risk of death due to any cause or due to cervical cancer between individuals with and without preexisting diagnosis of a mental disorder, respectively. No statistically significant interaction was found between a preexisting diagnosis of a mental disorder and calendar year of cancer diagnosis for overall mortality (*P* for interaction = .44) or for cervical cancer–specific mortality (*P* for interaction = .61).

## Discussion

In this cohort study of females in Sweden with a new cervical cancer diagnosis from 1978 to 2018, we found higher overall mortality in patients who had a preexisting diagnosis of a mental disorder compared with patients without a preexisting mental disorder diagnosis. The association remained after further adjustment for other characteristics, including cancer stage and tumor histologic features. Estimated 5-year survival of patients with cervical cancer increased from 1978 to 2018, regardless of the preexisting diagnosis of a mental disorder, but the relative differences in survival between women with and without a preexisting mental disorder diagnosis persisted. Unadjusted and partially adjusted analyses revealed an association between preexisting mental disorder and cancer-specific mortality, although these results were attenuated in the fully adjusted model.

Our findings are independent of cancer characteristics or other sociodemographic and medical covariables and are consistent with the existing literature on other cancer types.^31,32,33,34,35,36,37^ Compared with the fully adjusted model, the increased risk in overall mortality noted in the partially adjusted model suggested that the association was partially attributable to cancer and other characteristics, including FIGO stage, tumor histologic type, residential region, education level, and marital status at the time of cancer diagnosis. To our knowledge, only 1 study has reported that individuals with cervical cancer and disabilities, particularly severe mental disabilities, had a higher risk of death than those without such comorbidity.^25^ In our study, increased risk in overall mortality was primarily associated with mental disorders requiring inpatient care but not to mental disorders requiring only outpatient care, suggesting that the higher overall mortality was mainly associated with severe mental disorders. This interpretation is consistent with the direct association found for substance abuse, psychotic disorders, and depressive disorders but not for anxiety disorders and stress-related disorders. In terms of neurodevelopmental disorders, the number of outcomes was too small to draw any conclusion.

Findings of the present study suggest that the association between mental disorders and cervical cancer–specific mortality is partially attributable to delayed diagnosis and advanced tumor stage at diagnosis. The literature on mental disorders and cancer stage at diagnosis is conflicting.^31,34,37,38^ A meta-analysis found that a preexisting diagnosis of a mental illness was not associated with a greater odds of advanced cancer stage at diagnosis; however, when only population-based data from universal health care systems were included, there was an association.^31^ We found no statistically significant difference in FIGO stage at diagnosis between patients with or without a preexisting mental disorder. However, when examining FIGO distribution by severity of mental disorder, individuals who needed inpatient care for their mental disorder more often presented with a higher FIGO stage at cancer diagnosis. Although patients with or without a preexisting diagnosis of a mental disorder did not differ by mode of cervical cancer detection, patients with a preexisting mental disorder diagnosis were more likely to have squamous cell carcinoma than patients without a preexisting mental disorder diagnosis, suggesting there may be differences in causal factors. The lower survival among patients with cervical cancer and a preexisting mental health disorder might also arise due to differences in time to treatment, access to optimal treatment, quality of care, and treatment adherence.^39^ As in-depth information on treatment data was not available in the present study, future studies are warranted to address these possibilities.

To our knowledge, this is the first study to report on temporal trends in survival of patients with cervical cancer during a 40-year period by preexisting diagnosis of a mental disorder status. We observed a gradual increase in survival over time in both patients with and without a preexisting mental disorder, likely reflecting improvements in cancer screening, diagnosis, and treatment. For instance, detection of invasive cancer at screening entails a better prognosis than detection due to symptoms.^15^ Results of the present study are in line with Hemminki et al^40^ that showed an increase in 5-year survival from 60% to 69% from 1967 to 2016 among patients with cervical cancer in Sweden. Another study reported a similar increase in the 5-year age-standardized net survival of patients with cervical cancer from 1960 to 2014 in Sweden.^41^ Though comparisons of cancer survival between countries is challenging,^42^ improvement in cervical cancer survival has also been reported in other countries.^40,43^

### Strengths and Limitations

The main strength of the present study is that we included virtually all individuals with cervical cancer diagnosed from 1978 to 2018 in Sweden, with 40 years of follow-up. Another novelty of the study lies in the fact that, through record linkages, we were also able to obtain information on cancer, sociodemographic, and medical characteristics, as well as screening indicators, to understand the contribution of these factors to the association between a preexisting diagnosis of a mental disorder and mortality after a diagnosis of cervical cancer.

Nevertheless, this study has limitations. In-depth evaluations of mortality after cervical cancer in relation to specific mental disorders (eg, neurodevelopmental disorders) had insufficient statistical power and should be interpreted with caution. Furthermore, information on FIGO stage was available only from 2000 onward. Analyses including more clinical factors could therefore be carried out only within the subgroup of patients diagnosed with cervical cancer from 2002 to 2016. Finally, information on cause of death was obtained from the Swedish Cause of Death Register. Occasionally a primary cause of death cannot be established with certainty and, as a result, some deaths might be misclassified as cervical cancer–related. However, misclassification is most likely nondifferential and in fact might have biased the results toward null.

## Conclusions

This cohort study found that patients with cervical cancer who have a preexisting diagnosis of a mental disorder at the time of cancer diagnosis had lower overall and cervical cancer–specific survival than patients without a preexisting mental disorder diagnosis. These differences remained after adjustment for cancer characteristics and sociodemographics at the time of cancer diagnosis for overall survival but not for cancer-specific survival. These findings suggest that individuals with severe mental disorders should be considered a high-risk group in the tertiary prevention of cervical cancer.

## Notes

Supplement 1. **eFigure.** Details on Study Exclusions and Patient Cohorts Analyzed **eTable 1.** Pre-Cancer Mental Disorders and Corresponding ICD 8/9/10 Codes **eTable 2.** Distribution of FIGO Stage by Mental Disorder Severity **eTable 3.** Incidence Rates (IRs) and Hazard Ratios (HRs) With 95% CIs Comparing Overall Mortality in Patients with Cervical Cancer With vs Without a Preexisting Diagnosis of a Mental Disorder in the Patient Cohort of 2002-2016, by Type of Care for Mental Disorders **eTable 4.** Incidence Rates (IRs) and Hazard Ratios (HRs) With 95% CIs Comparing Cervical Cancer–Specific Mortality in Patients with Cervical Cancer With vs Without a Preexisting Diagnosis of a Mental Disorder in the Patient Cohort of 2002-2016, by Type of Care for Mental Disorders **eTable 5.** Five-Year Survival, Incidence Rates (IRs) and Hazard Ratios (HRs) of Overall Mortality in the Patient Cohort of 1978-2018, by Presence of a Preexisting Diagnosis of a Mental Disorder and Calendar Year of Cervical Cancer Diagnosis **eTable 6.** Five-Year Survival, Incidence Rates (IRs) and Hazard Ratios (HRs) of Cervical Cancer–Specific Mortality in the Patient Cohort of 1978-2018, by Presence of a Preexisting Diagnosis of a Mental and Calendar Year of Cervical Cancer Diagnosis

Supplement 2.
Data Sharing Statement
