# Supplementary material for: Overall and Cervical Cancer Survival in Patients With and Without Mental Disorders
Source: JAMA Netw Open. 2023 Sep 29;6(9):e2336213. doi: 10.1001/jamanetworkopen.2023.36213 (PMC10542737; doi:10.1001/jamanetworkopen.2023.36213)
Supplement: Supplement 1. — eFigure. Details on Study Exclusions and Patient Cohorts Analyzed eTable 1. Pre-Cancer Mental Disorders and Corresponding ICD 8/9/10 Codes eTable 2. Distribution of FIGO Stage by Mental Disorder Severity eTable 3. Incidence Rates (IRs) and Hazard Ratios (HRs) With 95% CIs Comparing Overall Mortality in Patients with Cervical Cancer With vs Without a Preexisting Diagnosis of a Mental Disorder in the Patient Cohort of 2002-2016, by Type of Care for Mental Disorders eTable 4. Incidence Rates (IRs) and Hazard Ratios (HRs) With 95% CIs Comparing Cervical Cancer–Specific Mortality in Patients with Cervical Cancer With vs Without a Preexisting Diagnosis of a Mental Disorder in the Patient Cohort of 2002-2016, by Type of Care for Mental Disorders eTable 5. Five-Year Survival, Incidence Rates (IRs) and Hazard Ratios (HRs) of Overall Mortality in the Patient Cohort of 1978-2018, by Presence of a Preexisting Diagnosis of a Mental Disorder and Calendar Year of Cervical Cancer Diagnosis eTable 6. Five-Year Survival, Incidence Rates (IRs) and Hazard Ratios (HRs) of Cervical Cancer–Specific Mortality in the Patient Cohort of 1978-2018, by Presence of a Preexisting Diagnosis of a Mental and Calendar Year of Cervical Cancer Diagnosis [file jamanetwopen-e2336213-s001.pdf]

Supplemental Online Content

Herweijer E, Wang J, Hu K, et al. Overall and cervical cancer survival in patients with and without mental disorders. *JAMA Netw Open*. 2023;6(9):e2336213. doi:10.1001/jamanetworkopen.2023.36213

eFigure 1. Details on Study Exclusions and Patient Cohorts Analyzed

eTable 1. Pre-Cancer Mental Disorders and Corresponding ICD 8/9/10 Codes

eTable 2. Distribution of FIGO Stage by Mental Disorder Severity

eTable 3. Incidence Rates (IRs) and Hazard Ratios (HRs) With 95% CIs Comparing Overall Mortality in Patients with Cervical Cancer With vs Without a Preexisting Diagnosis of a Mental Disorder in the Patient Cohort of 2002-2016, by Type of Care for Mental Disorders

eTable 4. Incidence Rates (IRs) and Hazard Ratios (HRs) With 95% CIs Comparing Cervical Cancer–Specific Mortality in Patients with Cervical Cancer With vs Without a Preexisting Diagnosis of a Mental Disorder in the Patient Cohort of 2002-2016, by Type of Care for Mental Disorders

eTable 5. Five-Year Survival, Incidence Rates (IRs) and Hazard Ratios (HRs) of Overall Mortality in the Patient Cohort of 1978-2018, by Presence of a Preexisting Diagnosis of a Mental Disorder and Calendar Year of Cervical Cancer Diagnosis

eTable 6. Five-Year Survival, Incidence Rates (IRs) and Hazard Ratios (HRs) of Cervical Cancer–Specific Mortality in the Patient Cohort of 1978-2018, by Presence of a Preexisting Diagnosis of a Mental and Calendar Year of Cervical Cancer Diagnosis

This supplemental material has been provided by the authors to give readers additional information about their work.

eFigure 1. Details on Study Exclusions and Patient Cohorts Analyzed

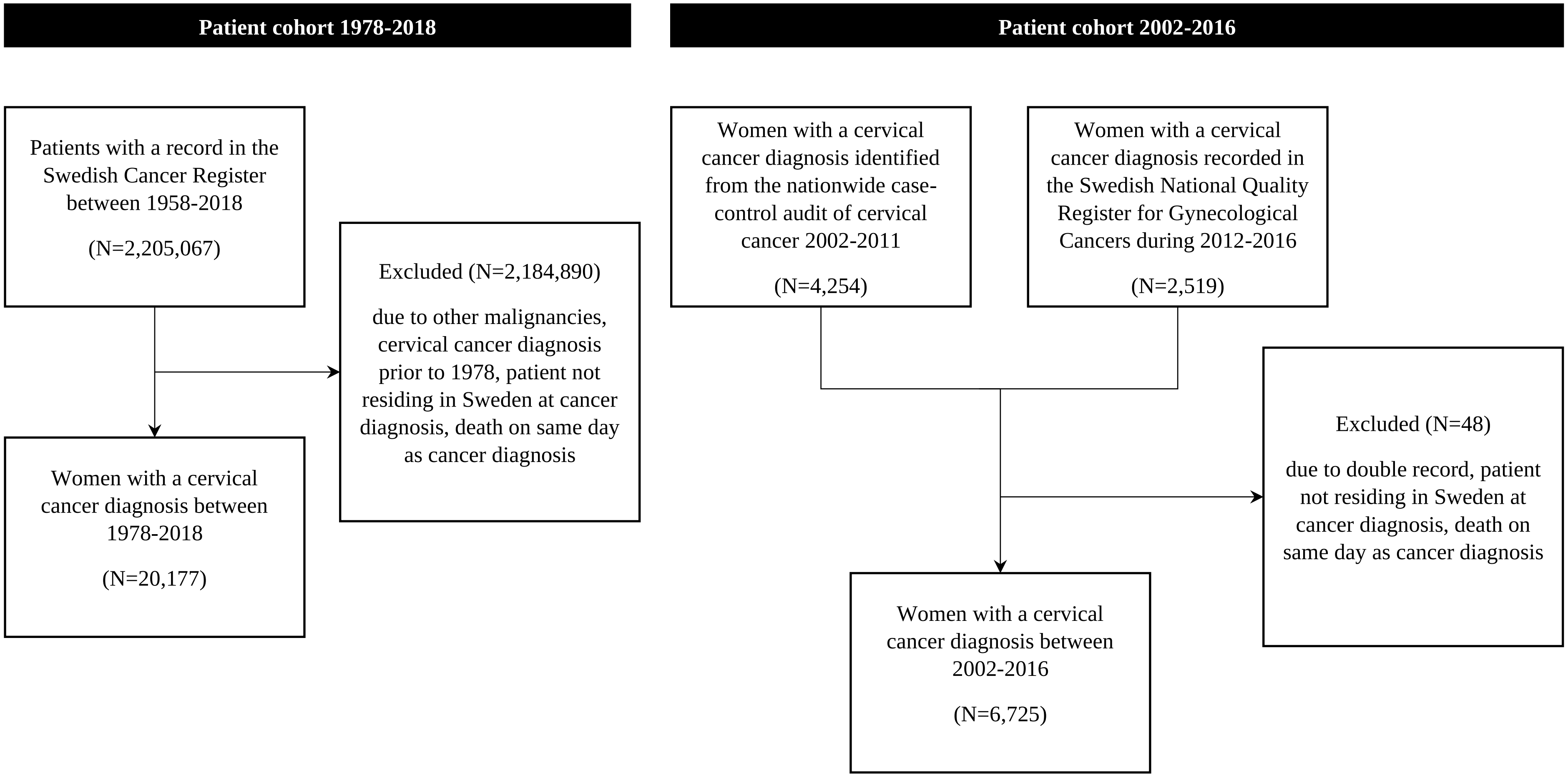

eTable 1. Pre-Cancer Mental Disorders and Corresponding ICD 8/9/10 Codes

Disorder	ICD-8 (1968-1986)	ICD-9 (1987-1996)	ICD-10 (1997-)
**Any mental disorder**	291, 295-315	291, 292, 295-319	F10-F99
**Psychiatric disorders**			
Substance abuse (not incl. tobacco and alcohol)	304	292, 303, 304, 305X	F10-F19 excl. F10, F17
Alcohol	291, 303	305B, 291	F10
Tobacco	-	305A	F17
Psychotic disorders (schizophrenia and non affective psychotic disorders)	295, 297, 298, 299	295, 297, 298	F20-F29
Depressive disorder	296.0, 296.2, 298.0, 300.4	296B, 296D, 298A, 300E, 311	F32-F33
Anxiety disorder	300.0, 300.2	300A, 300C	F40-F41
Stress-related disorders	307	308, 309	F43
**Neurodevelopmental disorders**			
Attention-deficit hyperactivity disorder	-	314	F90
Autism spectrum disorder	-	299	F84
Intellectual disability (mental retardation)	310-315	317-319	F70-F79

eTable 2. Distribution of FIGO Stage by Mental Disorder Severity

**Characteristics**	**No mental disorder**	**Any mental disorder requiring outpatient care alone**	**Any mental disorder requiring inpatient care**	**Total**	**P-value[1]**
**FIGO - n (%)**					
IA	1,272 (21.81)	91 (28.89)	114 (19.72)	1,477 (21.96)	<0.001
IB	2,257 (38.70)	133 (42.22)	197 (34.08)	2,587 (38.47)	
II+	2,237 (38.36)	84 (26.67)	256 (44.29)	2,577 (38.32)	
Unknown/missing	66 (1.13)	7 (2.22)	11 (1.90)	84 (1.90)	

Abbreviations: FIGO - The International Federation of Gynecology and Obstetrics

[1] P-value was based on Chi-square test.

eTable 3. Incidence Rates (IRs) and Hazard Ratios (HRs) With 95% CIs Comparing Overall Mortality in Patients with Cervical Cancer With vs Without a Preexisting Diagnosis of a Mental Disorder in the Patient Cohort of 2002-2016, by Type of Care for Mental Disorders

Groups[1]	Deaths	Person-years	Crude IR per 1000 person-years (95%CI)	Partially adjusted HR (95%CI)[2]	Fully adjusted HR (95%CI)[3]
**Any mental disorder**					
No	2128	42148	50.49 (48.39-52.68)	Ref.	Ref.
Yes – outpatient care alone	71	1962	36.18 (28.67-45.65)	1.08 (0.85-1.37)	1.03 (0.81-1.32)
Yes – inpatient care	267	3269	81.68 (72.45-92.09)	1.39 (1.23-1.58)	1.24 (1.09-1.42)
**By type of psychiatric disorders**					
Substance abuse (incl. tobacco and alcohol)					
No	2329	45684	50.98 (48.95-53.09)	Ref.	Ref.
Yes – outpatient care alone	15	288	52.06 (31.38-86.35)	1.43 (0.87-2.35)	1.29 (0.77-2.16)
Yes – inpatient care	122	1407	86.69 (72.59-103.52)	1.53 (1.27-1.83)	1.27 (1.05-1.53)
Psychotic disorders[4]					
No	2409	46930	51.33 (49.32-53.42)	Ref.	Ref.
Yes – outpatient care alone	3	63	47.52 (15.32-147.32)	1.24 (0.40-3.86)	0.94 (0.28-3.15)
Yes – inpatient care	54	386	139.82 (107.09-182.56)	1.78 (1.36-2.34)	1.53 (1.15-2.04)
Depressive disorder					
No	2330	45081	51.68 (49.63-53.83)	Ref.	Ref.
Yes – outpatient care alone	42	1173	35.81 (26.47-48.46)	1.10 (0.81-1.50)	1.04 (0.76-1.42)
Yes – inpatient care	94	1126	83.49 (68.21-102.19)	1.35 (1.10-1.66)	1.38 (1.11-1.71)
Anxiety disorder					
No	2377	45542	52.19 (50.14-54.33)	Ref.	Ref.
Yes – outpatient care alone	55	1118	49.2 (37.77-64.08)	1.34 (1.02-1.76)	1.32 (1.00-1.74)
Yes – inpatient care	34	720	47.24 (33.75-66.11)	0.93 (0.66-1.31)	0.92 (0.64-1.30)

Stress-related disorders					
No	2396	45717	52.41 (50.35-54.55)	Ref.	Ref.
Yes – outpatient care alone	20	773	25.89 (16.70-40.13)	0.85 (0.55-1.32)	0.87 (0.55-1.36)
Yes – inpatient care	50	890	56.17 (42.57-74.11)	1.12 (0.85-1.48)	0.99 (0.74-1.32)

Abbreviations: IR – incidence rate, CI – 95% confidence interval, HR – hazard ratio, ref – reference category

[1] Results by type of neurodevelopmental disorder are not shown due to insufficient power.

[2] Adjusted for age and calendar year of diagnosis (continuous)

[3] Adjusted for age, calendar year of diagnosis (continuous), FIGO stage, tumor histology, residing region, educational level, and marital status

[4] Psychotic disorders including schizophrenia and non-affective psychotic disorders

eTable 4. Incidence Rates (IRs) and Hazard Ratios (HRs) With 95% CIs Comparing Cervical Cancer–Specific Mortality in Patients with Cervical Cancer With vs Without a Preexisting Diagnosis of a Mental Disorder in the Patient Cohort of 2002-2016, by Type of Care for Mental Disorders

Groups[1]	Deaths	Person-years	Crude IR per 1000 person-years (95%CI)	Partially adjusted HR (95%CI)[2]	Fully adjusted HR (95%CI)[3]
**Any mental disorder**					
No	1401	42148	33.24 (31.54-35.03)	Ref.	Ref.
Yes – outpatient care alone	51	1962	25.99 (19.75-34.19)	0.98 (0.74-1.30)	0.94 (0.70-1.25)
Yes – inpatient care	173	3269	52.92 (45.6-61.43)	1.33 (1.14-1.56)	1.19 (1.01-1.40)
**By type of psychiatric disorders**					
Any substance abuse (incl. tobacco and alcohol)					
No	1534	45684	33.58 (31.94-35.3)	Ref.	Ref.
Yes – outpatient care alone	13	288	45.11 (26.2-77.7)	1.58 (0.93-2.68)	1.38 (0.79-2.41)
Yes – inpatient care	78	1407	55.42 (44.39-69.2)	1.41 (1.12-1.77)	1.19 (0.94-1.50)
Psychotic disorders[4]					
No	1590	46930	33.88 (32.25-35.59)	Ref.	Ref.
Yes – outpatient care alone	2	63	31.68 (7.92-126.66)	1.09 (0.27-4.37)	0.76 (0.18-3.24)
Yes – inpatient care	33	386	85.45 (60.75-120.19)	1.70 (1.20-2.40)	1.44 (1.01-2.05)
Depressive disorder					
No	1538	45081	34.12 (32.45-35.86)	Ref.	Ref.
Yes – outpatient care alone	29	1173	24.73 (17.18-35.58)	0.96 (0.66-1.39)	0.93 (0.64-1.35)
Yes – inpatient care	58	1126	51.51 (39.83-66.63)	1.24 (0.95-1.61)	1.28 (0.97-1.67)
Anxiety disorder					
No	1568	45542	34.43 (32.77-36.18)	Ref.	Ref.
Yes – outpatient care alone	41	1118	36.67 (27.00-49.81)	1.24 (0.90-1.69)	1.24 (0.90-1.71)
Yes – inpatient care	16	720	22.23 (13.62-36.28)	0.64 (0.39-1.04)	0.66 (0.39-1.10)
Stress-related disorders					
No	1577	45717	34.49 (32.83-36.24)	Ref.	Ref.

Yes – outpatient care alone	15	773	19.42 (11.71-32.21)	0.77 (0.46-1.29)	0.80 (0.48-1.34)
Yes – inpatient care	33	890	37.07 (26.36-52.15)	1.07 (0.76-1.51)	0.95 (0.67-1.36)

Abbreviations: IR – incidence rate, CI – 95% confidence interval, HR – hazard ratio, ref – reference category

[1] Results by type of neurodevelopmental disorder are not shown due to insufficient power.

[2] Adjusted for age and calendar year of diagnosis (continuous)

[3] Adjusted for age, calendar year of diagnosis (continuous), FIGO stage, tumor histology, residing region, education level, and marital status

[4] Psychotic disorders including schizophrenia and non-affective psychotic disorders

eTable 5. Five-Year Survival, Incidence Rates (IRs) and Hazard Ratios (HRs) of Overall Mortality in the Patient Cohort of 1978-2018, by Presence of a Preexisting Diagnosis of a Mental Disorder and Calendar Year of Cervical Cancer Diagnosis

**Groups**	**Deaths**	**Person-years**	**5-year survival (%)**	**Crude IR per 1000 person-years (95%CI)**	**Fully adjusted HR (95%CI)[a]**
**Any mental disorder**					
No	9995	221754	66.17	45.07 (44.2-45.96)	Ref.
Yes	704	9107	45.17	77.3 (71.8-83.23)	1.53 (1.42-1.66)
**Any mental disorder stratified by calendar period of diagnosis**					
1978-1989					
No	4563	101621	61.16	44.9 (43.62-46.22)	Ref.
Yes	171	2390	54.02	71.54 (61.58-83.1)	1.58 (1.36-1.85)
1990-1999					
No	2552	62099	64.97	41.1 (39.53-42.72)	Ref.
Yes	196	2658	52.36	73.75 (64.11-84.83)	1.64 (1.42-1.90)
2000-2009					
No	1860	40598	68.08	45.82 (43.78-47.95)	Ref.
Yes	183	2346	52.06	78.01 (67.49-90.17)	1.39 (1.19-1.62)
2010-2018					
No	1020	17439	73.88	58.49 (55.01-62.19)	Ref.
Yes	154	1713	63.12	89.89 (76.76-105.28)	1.52 (1.28-1.80)
					P^b=0.44

Abbreviations: IR – incidence rate, CI – 95% confidence interval, HR – hazard ratio, ref – reference category

[a] Adjusted for age, tumor histology, residing region, and calendar year of diagnosis (5-year categories)

[b] Likelihood ratio test to test for interaction between mental disorder status and calendar year of diagnosis

eTable 6. Five-Year Survival, Incidence Rates (IRs) and Hazard Ratios (HRs) of Cervical Cancer–Specific Mortality in the Patient Cohort of 1978-2018, by Presence of a Preexisting Diagnosis of a Mental and Calendar Year of Cervical Cancer Diagnosis

Groups	Deaths	Person-years	5-year survival (%)	Crude IR per 1000 person-years (95%CI)	Fully adjusted HR (95%CI)[a]
**Any mental disorder**					
No	5083	221756	74.90	22.92 (22.3-23.56)	Ref.
Yes	370	9107	68.82	40.63 (36.69-44.99)	1.34 (1.20-1.49)
**Any mental disorder stratified by calendar period of diagnosis**					
1978-1989					
No	2001	101621	70.58	19.69 (18.85-20.57)	Ref.
Yes	74	2390	66.60	30.96 (24.65-38.88)	1.36 (1.08-1.72)
1990-1999					
No	1234	62099	75.02	19.87 (18.79-21.01)	Ref.
Yes	78	2658	68.72	29.35 (23.51-36.64)	1.18 (.94-1.49)
2000-2009					
No	1083	40598	76.85	26.68 (25.13-28.31)	Ref.
Yes	114	2346	63.18	48.6 (40.45-58.39)	1.45 (1.19-1.76)
2010-2018					
No	765	17439	79.52	43.87 (40.87-47.09)	Ref.
Yes	104	1713	74.36	60.71 (50.09-73.57)	1.33 (1.09-1.64)
					P^b=0.61

Abbreviations: IR – incidence rate, CI – 95% confidence interval, HR – hazard ratio, ref – reference category

[a] Adjusted for age, tumor histology, residing region, and calendar year of diagnosis (5-year categories)

[b] Likelihood ratio test to test for interaction between mental health status and calendar year of diagnosis
