# Supplementary material for: Overall and Cervical Cancer Survival in Patients With and Without Mental Disorders
Source: JAMA Netw Open. 2023 Sep 29;6(9):e2336213. doi: 10.1001/jamanetworkopen.2023.36213 (PMC10542737; doi:10.1001/jamanetworkopen.2023.36213)
Supplement: Supplement 2. — Data Sharing Statement [file jamanetwopen-e2336213-s002.pdf]

Data Sharing Statement

Herweijer. Overall and Cervical Cancer Survival in Patients With and Without Mental Disorders. *JAMA Netw Open*. Published September 29, 2023. doi:10.1001/jamanetworkopen.2023.36213

Data
Data available: No

Additional Information
Explanation for why data not available: The raw datasets are not available for sharing because of privacy policies and regulations in Sweden. Additional data, analysis coding, and aspects of our protocol might be available by request to the corresponding author, provided that applicable policies and regulations are followed.
